# The receptor protein tyrosine phosphatase PTPRJ negatively modulates the CD98hc oncoprotein in lung cancer cells

**DOI:** 10.18632/oncotarget.25101

**Published:** 2018-05-04

**Authors:** Sabrina D’Agostino, Delia Lanzillotta, Mariaconcetta Varano, Cirino Botta, Antonio Baldrini, Anna Bilotta, Stefania Scalise, Vincenzo Dattilo, Rosario Amato, Eugenio Gaudio, Francesco Paduano, Camillo Palmieri, Rodolfo Iuliano, Nicola Perrotti, Cesare Indiveri, Alfredo Fusco, Marco Gaspari, Francesco Trapasso

**Affiliations:** ^1^ Dipartimento di Medicina Sperimentale e Clinica, University Magna Græcia, Campus “S. Venuta”, Catanzaro, Italy; ^2^ Dipartimento di Scienze della Salute, University Magna Græcia, Campus “S. Venuta”, Catanzaro, Italy; ^3^ Lymphoma and Genomics Research Program, IOR Institute of Oncology Research, Bellinzona, Switzerland; ^4^ Tecnologica Research Institute, Biomedical Section, Crotone, Italy; ^5^ Dipartimento di Biologia, Ecologia, Scienze Della Terra (DiBEST), Unit of Biochemistry and Molecular Biotechnology, University of Calabria, Arcavacata di Rende, Italy; ^6^ Istituto di Endocrinologia e Oncologia Sperimentale, CNR c/o Dipartimento di Medicina Molecolare e Biotecnologie Mediche, University “Federico II” of Napoli, Napoli, Italy

**Keywords:** protein tyrosine phosphatase, PTPRJ, CD98hc, proteasomal degradation, lung cancer

## Abstract

PTPRJ, a receptor protein tyrosine phosphatase strongly downregulated in human cancer, displays tumor suppressor activity by negatively modulating several proteins involved in proliferating signals. Here, through a proteomic-based approach, we identified a list of potential PTPRJ-interacting proteins and among them we focused on CD98hc, a type II glycosylated integral membrane protein encoded by *SLC3A2*, corresponding to the heavy chain of a heterodimeric transmembrane amino-acid transporter, including LAT1. CD98hc is widely overexpressed in several types of cancers and contributes to the process of tumorigenesis by interfering with cell proliferation, adhesion, and migration. We first validated PTPRJ-CD98hc interaction, then demonstrated that PTPRJ overexpression dramatically reduces CD98hc protein levels in A549 lung cancer cells. In addition, following to the treatment of PTPRJ-transduced cells with MG132, a proteasome inhibitor, CD98hc levels did not decrease compared to controls, indicating that PTPRJ is involved in the regulation of CD98hc proteasomal degradation. Moreover, PTPRJ overexpression combined with CD98hc silencing consistently reduced cell proliferation and triggered apoptosis of lung cancer cells. Interestingly, by interrogating the *can Evolve* database, we observed an inverse correlation between *PTPRJ* and *SLC3A2* gene expression. Indeed, the non-small cell lung cancers (NSCLCs) of patients showing a short survival rate express the lowest and the highest levels of *PTPRJ* and *SLC3A2*, respectively. Therefore, the results reported here contribute to shed lights on PTPRJ signaling in cancer cells: moreover, our findings also support the development of a novel anticancer therapeutic approach by targeting the pathway of PTPRJ that is usually downregulated in highly malignant human neoplasias.

## INTRODUCTION

PTPRJ, a member of the protein tyrosine phosphatases family with tumor suppressor activity, is downregulated in several cancer tissues and cell lines and its restoration blocks their proliferation [1–3]. Other than promoter hypermethylation [4] and loss of heterozygosity (LOH), observed in colon, lung, mammary, and thyroid tumors [5–7], we have also previously shown that *PTPRJ* is a target of miR-328. Indeed, miR-328 expression leads to *PTPRJ* downregulation through increasing cell proliferation of cancer cell lines such as HeLa and SKBr3 [8]. An inverse correlation between miR-328 and *PTPRJ* mRNA levels has also been assessed in hepatocellular carcinoma, where PTPRJ expression negatively correlates with the progression of this malignancy [9]. However, even though specific *Ptprj* polymorphic alleles predispose to colon cancer [5], no spontaneous tumors have been described in *Ptprj*^-/-^ mice [10].

Our group also demonstrated that virus-mediated PTPRJ overexpression is effective in preclinical models of both thyroid and pancreatic cancer [11, 12], suggesting that *PTPRJ* is a proof-of-principle therapeutic gene. This assumption was supported not only by the anti-proliferative effects obtained through PTPRJ stimulation by a monoclonal antibody [13] but also by the discovery of synthetic PTPRJ agonist peptides able to reduce the extent of MAPK phosphorylation and, conversely, to increase cell cycle inhibitor p27^Kip1^ protein levels; these PTPRJ agonist peptides also reduce both cancer cell proliferation and tubulogenesis as well as trigger apoptosis of cancer cells [14, 15]. Nowadays, two PTPRJ biological ligands have been identified: heparansulfate proteoglycan Syndecan-2 (S2ED) and Thrombospondin-1; S2ED-bound PTPRJ mediates cell adhesion by modulating β1 integrin-mediated adhesion and cytoskeletal organization while TSP1 binding increases PTPRJ activity [16, 17].

The biochemical pathways negatively regulated by PTPRJ have been only partly clarified; several studies report an inhibitory effect of PTPRJ on several key factors of the mitogenic signaling; in fact, PTPRJ dephosphorylates several receptor tyrosine kinases such as PDGFR [18], HGFR [19], RET [20], EGFR [21], and VEGFR [22] as well as downstream cytosolic transducers such as MAPK [23], thus inhibiting the mitogenic signals driven by them.

Here, in the attempt to further shed lights on the PTPRJ protein network and its role in cancer, we identified several novel putative candidate PTPRJ protein partners by using a proteomic-based approach. Among the listed proteins, we focused on CD98hc, a protein encoded by the *SLC3A2* gene. We first demonstrated PTPRJ-CD98hc association, then that PTPRJ overexpression leads to CD98hc decreased protein levels through a proteasomal-dependent degradation pathway apparently resulting in a reduced extent of both cell proliferation and migration. Moreover, PTPRJ and CD98hc inverse correlation, assessed by interrogating *canEvolve* database in a large number of lung cancer patients, not only proposes these proteins as candidate biomarkers of tumor aggressiveness but also encourages the development of novel targeted therapies for the treatment of cancer.

## RESULTS

### Identification and validation of CD98hc as a PTPRJ protein partner

**Figure 1:**
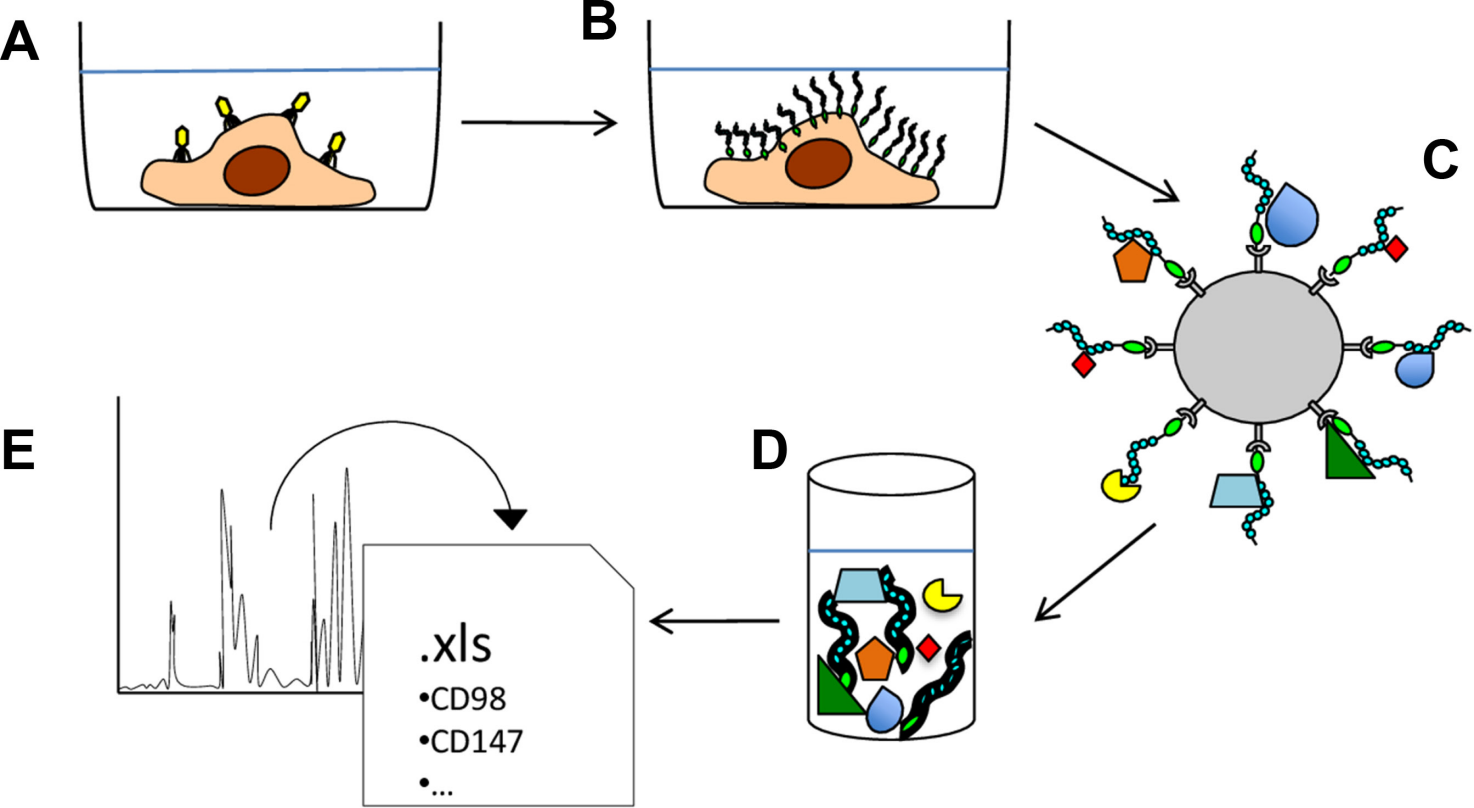
Schematic representation of the experimental approach used to identify PTPRJ-interacting proteins (**A**) A549 lung cancer cells were transduced with a recombinant adenovirus carrying a cDNA coding for PTPRJ fused to a His6-tag. (**B**) transduced A549 cells were lysed and enriched membrane proteins were extracted. (**C**) PTPRJ-His6 and its candidate interacting-proteins were isolated through poly(His)-avid magnetic beads; (**D**) beads-bound proteins were eluted and (**E**) analyzed for peptides identification by mass spectrometry.

To isolate novel PTPRJ-interacting proteins, a recombinant adenovirus, coding for a histidine six-tagged PTPRJ, was used to infect recipient A549 cells as described [14]. Briefly, seventy-two hours after infection, A549-transduced cells were lysed and cell membranes enriched to maximize the purification of mature integral proteins. Recombinant PTPRJ-His6 protein along with its potentially interacting proteins were isolated from membrane extracts by using poly(His)-avid magnetic beads and processed by mass spectrometry (Figure 1). A large number of proteins were identified (699), as listed in Supplementary Table 1. Among them, 47 proteins were significantly enriched in the PTPRJ-His6 sample (*p*-value <0.01, fold change >2). These proteins are listed in Table 1, whereas their Gene Ontology (GO) enrichment analysis is reported in Supplementary Table 2. Among the candidate PTPRJ-binding proteins, we focused on CD98hc.

**Table 1:** List of significantly enriched proteins in PTPRJ-His6 pull-down with respect of the control experiment

Uniprot Accession	Protein	Gene	Sequence Coverage	Unique Peptides	Peptides	MEAN M:L	*p*-value
P20020	Plasma membrane calcium-transporting ATPase 1	ATP2B1	4.85	4	4	8.13	0.00
H7C5Q2	Acyl-coenzyme A thioesterase 9, mitochondrial (Fragment)	ACOT9	13.26	2	2	8.13	0.00
Q12913	Receptor-type tyrosine-protein phosphatase eta	PTPRJ	31.79	39	39	7.89	0.00
A0A0A0MR02	Voltage-dependent anion-selective channel protein 2 (Fragment)	VDAC2	12.77	3	3	7.74	0.00
O95297	Myelin protein zero-like protein 1	MPZL1	14.13	3	3	7.43	0.00
P67936	Tropomyosin alpha-4 chain	TPM4	25.81	5	8	6.90	0.00
Q9H330	Transmembrane protein 245	TMEM245	2.31	2	2	6.54	0.00
P61019	Ras-related protein Rab-2A	RAB2A	19.34	3	3	6.53	0.00
Q9UH99	SUN domain-containing protein 2	SUN2	3.77	2	2	5.86	0.01
J3KNL5	GRAM domain-containing protein 1B	GRAMD1B	4.07	2	2	5.71	0.00
O75306	NADH dehydrogenase [ubiquinone] iron-sulfur protein 2, mitochondrial	NDUFS2	7.56	3	3	5.62	0.00
P42166	Lamina-associated polypeptide 2, isoform alpha	TMPO	2.88	2	2	5.40	0.01
Q8NBU5	ATPase family AAA domain-containing protein 1	ATAD1	18.56	5	5	5.35	0.00
J3KTA4	Probable ATP-dependent RNA helicase DDX5	DDX5	9.28	5	6	5.17	0.00
F5H004	Ras-related protein Rap-1b (Fragment)	RAP1B	22.83	4	4	5.08	0.00
D6RBN9	RELT-like protein 1 (Fragment)	RELL1	25.51	3	3	4.79	0.00
Q96ER9	Coiled-coil domain-containing protein 51	CCDC51	14.11	5	5	4.64	0.00
Q8N766	ER membrane protein complex subunit 1	EMC1	5.34	4	4	4.21	0.01
E9PB51	RNA-binding protein 4 (Fragment)	RBM4	15	4	4	3.88	0.00
P51148	Ras-related protein Rab-5C	RAB5C	21.3	4	4	3.73	0.01
X6RFL8	Ras-related protein Rab-14 (Fragment)	RAB14	24.86	5	5	3.62	0.00
P35580	Myosin-10	MYH10	10.17	7	17	3.59	0.00
P04083	Annexin A1	ANXA1	14.16	4	4	3.54	0.01
Q92896	Golgi apparatus protein 1	GLG1	19.42	22	22	3.47	0.00
P18031	Tyrosine-protein phosphatase non-receptor type 1	PTPN1	15.17	6	6	3.36	0.01
P14618	Pyruvate kinase PKM	PKM	47.46	21	21	3.20	0.00
O94826	Mitochondrial import receptor subunit TOM70	TOMM70A	9.05	5	5	3.15	0.00
P49257	Protein ERGIC-53	LMAN1	20.2	10	11	3.13	0.00
P21980	Protein-glutamine gamma-glutamyltransferase 2	TGM2	8.3	6	6	2.95	0.00
P61011	Signal recognition particle 54 kDa protein	SRP54	17.86	7	7	2.94	0.00
P46940	Ras GTPase-activating-like protein IQGAP1	IQGAP1	14.3	17	17	2.94	0.00
P27105	Erythrocyte band 7 integral membrane protein	STOM	52.43	15	15	2.93	0.00
P35579	Myosin-9	MYH9	37.24	62	73	2.89	0.00
P14625	Endoplasmin	HSP90B1	16.06	11	11	2.84	0.01
F5GZS6	4F2 cell-surface antigen heavy chain (CD82hc)	SLC3A2	23.04	13	13	2.80	0.00
A0A0A0MS51	Gelsolin	GSN	10.56	7	7	2.80	0.01
P08107	Heat shock 70 kDa protein 1A/1B	HSPA1A	15.76	10	11	2.71	0.00
P05026	Sodium/potassium-transporting ATPase subunit beta-1	ATP1B1	17.82	4	4	2.71	0.01
P36542	ATP synthase subunit gamma, mitochondrial	ATP5C1	25.17	8	8	2.65	0.00
O94979	Protein transport protein Sec31A	SEC31A	7.05	8	8	2.64	0.00
P27824	Calnexin	CANX	15.2	8	8	2.47	0.01
Q13813	Spectrin alpha chain, non-erythrocytic 1	SPTAN1	4.94	9	9	2.47	0.00
P08670	Vimentin	VIM	21.03	7	10	2.45	0.01
P25705	ATP synthase subunit alpha, mitochondrial	ATP5A1	21.7	11	11	2.40	0.00
P19105	Myosin regulatory light chain 12A	MYL12A	34.5	5	5	2.39	0.00
P31327	Carbamoyl-phosphate synthase [ammonia], mitochondrial	CPS1	17.47	23	25	2.28	0.00
H7C463	MICOS complex subunit MIC60 (Fragment)	IMMT	15.66	10	10	2.16	0.01

Section of potential PTPRJ-interacting protein list obtained by mass spectrometry analysis. Uniprot Accession numbers along with a brief description of protein functions were included in the table. “Sequence coverage” value corresponds to the total quantity of each protein present in the samples; “Unique Peptides” indicates the number of exclusive peptides found for each protein group while “Peptides” correlates with the total number of peptides assigned to each protein group. Medium/light ratio (Mean M:L) represents the fold change between eluted sample and eluted control. For more details see Supplementary Information 1.

Several findings prompted us to investigate in further detail the PTPRJ-CD98hc interaction. CD98hc heterodimerize with different light chains, thus forming amino-acidic transporters in cell membranes [24]. CD98hc constitutive deletion in mice results in early embryonic death [25], while CD98hc overexpression induced tumorigenesis in the gastrointestinal epithelium by stimulating cell proliferation [26]. Moreover, CD98hc overexpression, widely occurring in human cancer, correlates with dismal prognosis [27, 28]. Although the exact contribution of CD98hc to tumorigenesis is still not fully understood, it has been shown that it is involved in cell proliferation by mTOR pathway activation through the intracellular amino-acids transport [29]; recently, it has been reported that CD98hc cytoplasmatic domain also induces proliferation of renal epithelial cells by activating Erk and p38 MAPK signaling [30]. On the other hand, CD98hc/β-integrin interaction was shown to be important in modulating cell spreading, migration [31], transformation [32], and survival [33].

**Figure 2:**
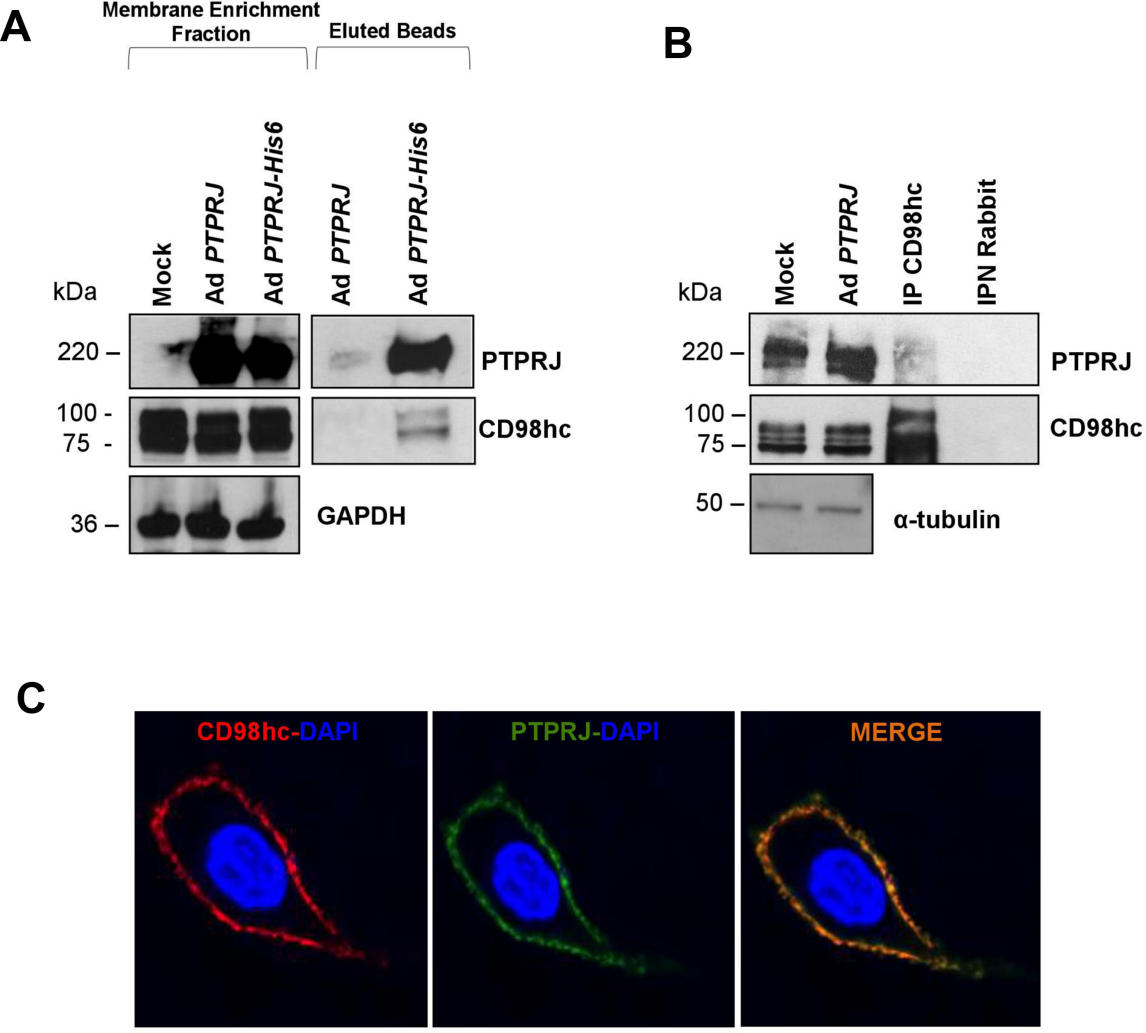
PTPRJ interacts and colocalizes with CD98hc (**A**) A549 lung cancer cells were transduced either with Ad *PTPRJ* (used as a control) or Ad *PTPRJ*-His6. Seventy-two hours after infection, cells were lysed and cell membranes enriched. Mature integral fusion protein PTPRJ-His6 were purified through poly(his)-avid magnetic beads, loaded on a polyacrylamide gel, transferred to nitrocellulose filter, and probed with CD98hc antibody. (**B**) A549 lung cancer cells were transduced with a recombinant Ad *PTPRJ*. Twenty-four hours after infection, cells were lysed and proteins extracted. Total protein lysates were immunoprecipitated with IgG and CD98hc. Proteins were loaded on a polyacrylamide gel, transferred to nitrocellulose filter, and probed with PTPRJ antibody. IgG was used to demonstrate that without CD98hc, PTPRJ is absent in the purified protein extract. (**C**) A549 cells were transduced with Ad *PTPRJ* at MOI30. Twenty-four hours later, A549 cells were incubated with PTPRJ mAb and CD98hc mAb; afterwards, secondaries rabbit-633 Ab (red) and mouse-PE Ab (green) were added to stain cells, as described in Methods, and observed by confocal microscopy. Merge of the images indicated the colocalization of PTPRJ and CD98hc.

First, we validated the interaction between recombinant His6-tagged PTPRJ protein and endogenous CD98hc in A549 lung cancer cells by co-immunoprecipitation experiments; no CD98hc protein was observed in the same assay performed with the PTPRJ protein used as a control (Figure 2A). Next, we also demonstrated that endogenous CD98hc co-immunoprecipitated ectopic PTPRJ protein overexpressed in A549 cells twenty-four hours after infection with a recombinant adenovirus carrying a wild-type *PTPRJ* cDNA at MOI30 (Figure 2B).

Finally, to investigate PTPRJ and CD98hc subcellular localization, A549 cells were transduced with Ad *PTPRJ* at MOI30; twenty-four hours later, both proteins clearly colocalized at the plasma membrane level (Figure 2C).

### PTPRJ overexpression negatively modulates CD98hc tyrosine-phosphorylation and decreases its protein levels

**Figure 3:**
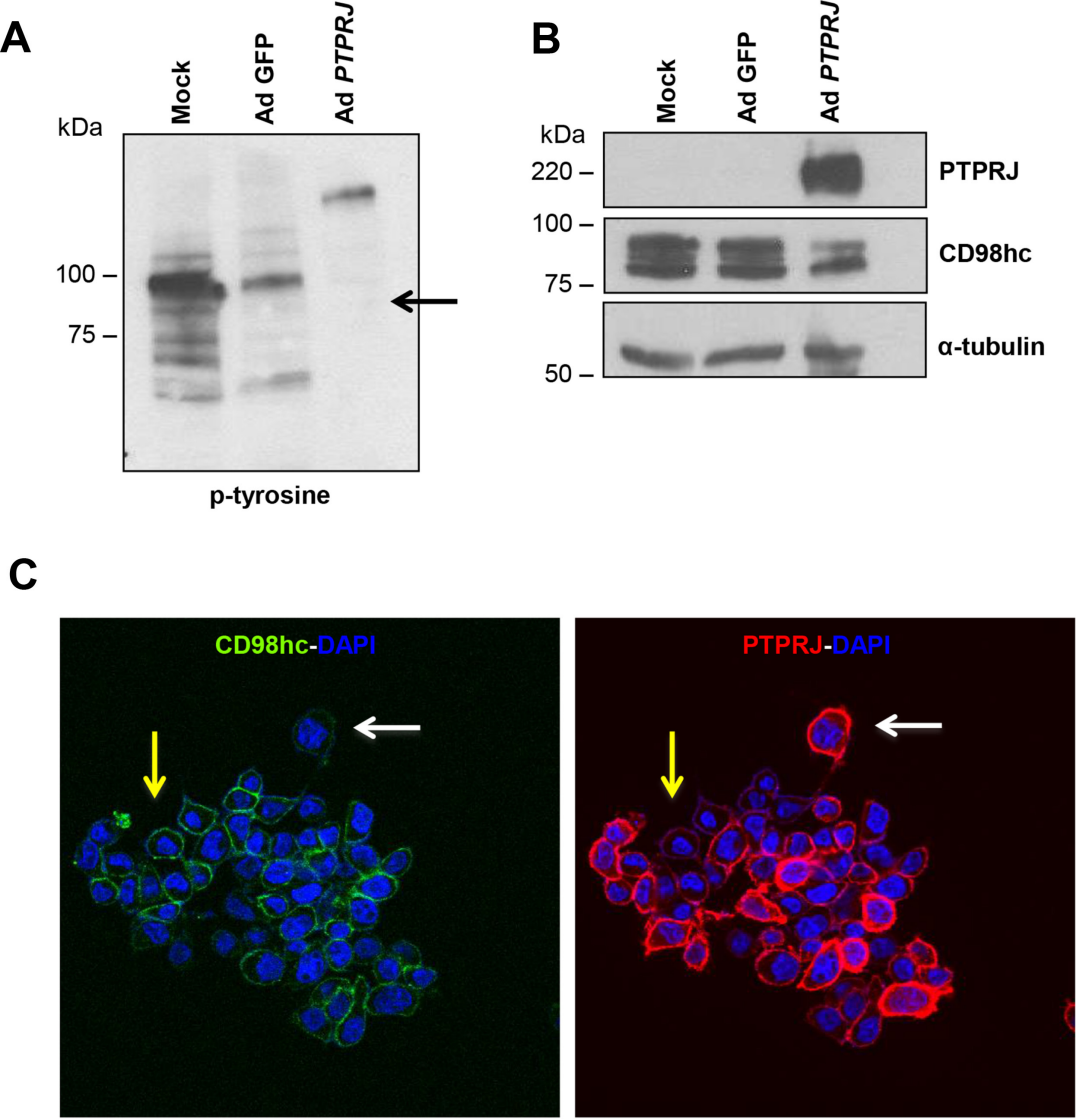
PTPRJ overexpression dephosphorylates CD98hc and downregulates its protein levels (**A, B**) A549 cells were seeded in 100 mm culture dishes and, after twenty-four hours, transduced by a recombinant Ad *PTPRJ* at MOI50. Forty-eight hours later, cells were lysed and extracted proteins loaded on polyacrylamide gel, transferred to nitrocellulose filter, and analyzed by Western blot through CD98hc, PTPRJ and p-tyrosine antibodies. γ-tubulin was used to normalize protein loading. (**C**) An immunofluorescence was also performed. A549 cells were transduced with Ad *PTPRJ* at MOI50. Forty-eight hours later, A549 cells were incubated with PTPRJ mAb and CD98hc mAb; afterwards, secondary rabbit-633 Ab (green) and mouse-PE Ab (red) were added to stain cells, as described in Materials and Methods, and observed by confocal microscopy. CD98hc levels decrease with PTPRJ overexpression (white arrow) in contrast with a lower expression of PTPRJ where CD98hc levels do not change (yellow arrow).

Since PTPRJ interacts with CD98hc, based on the intrinsic biochemical activity of this protein tyrosine phosphatase, we investigated CD98hc protein phosphorylation status. Following to PTPRJ overexpression in A549 cells, CD98hc tyrosine-phosphorylation extent and CD98hc protein levels were investigated. A549 cells were infected at MOI50 with Ad *PTPRJ*. Forty-eight hours later, we observed a highly significant reduction of CD98hc tyrosine-phosphorylation (Figure 3A). Conversely, CD98hc tyrosine-phosphorylation status was unchanged either in untreated or in Ad GFP-infected A549 cells. Interestingly, we noticed that CD98hc protein levels were strongly affected by PTPRJ overexpression compared to mock-infected cells (Figure 3B).

To confirm CD98hc downregulation following to PTPRJ overexpression, forty-eight hours after infection with Ad *PTPRJ* at MOI50, A549 cells were also investigated by immunofluorescence through confocal microscopy. Results, reported in Figure 3C, show a dramatic CD98hc decrease staining at plasma membrane level in cells showing the highest PTPRJ overexpression.

### PTPRJ induces CD98hc ubiquitin-mediated proteasome-dependent degradation

**Figure 4:**
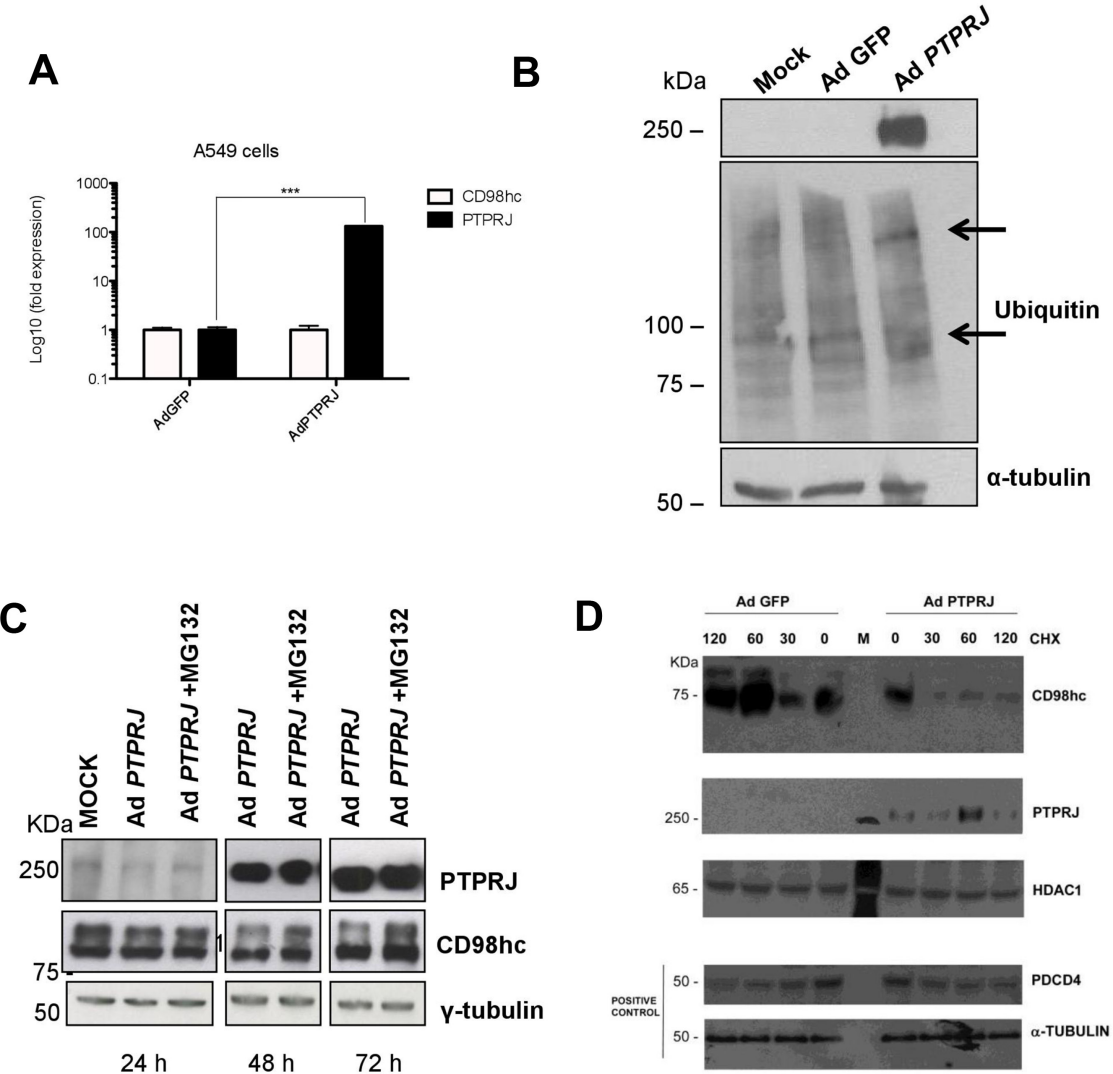
PTPRJ mediates CD98hc stability and proteasome degradation (**A**) A549 cells were seeded in 100 mm culture dishes and, twenty-four hours later, transduced by a recombinant Ad *PTPRJ* at MOI50. After seventy-two hours, cells were lysed and retro-transcribed mRNA was analyzed by qRT-PCR using specific primers amplifying both CD98hc and PTPRJ. Values were normalized to HPRT RNA levels. ^*^P < 0.05. (**B**) A549 cells were transduced with Ad *PTPRJ* at MOI50; cells were lysed and extracted proteins loaded on polyacrylamide gel, transferred to nitrocellulose filter, and analyzed by Western blot through PTPRJ and ubiquitin antibodies. α-tubulin was used to normalize protein loading. (**C**) PTPRJ-overexpressing A549 cells were lysed after a 6 h treatment with 10 microM MG132, a proteasome inhibitor. The protein extract was analyzed by Western blot analysis evaluating CD98hc levels. γ-tubulin antibody was used to normalize. (**D**) A549 cells were seeded in 100 mm culture dishes. The cells were transduced by a recombinant Ad *PTPRJ* or Ad GFP as a control both at MOI50. Fourty-eight hours after infection, cells were treated with cycloeximide 100 μg/mL at different time points. Total proteins were extracted, loaded on polyacrylamide gel and stained with anti CD98hc, PTPRJ and Pdcd4. Equal loading was verified by tubulin and HDAC.

To better understand the mechanisms of CD98hc protein downregulation in PTPRJ-overexpressing A549 cancer cells, we first evaluated CD98hc mRNA expression by real time-PCR. As shown in Figure 4A, no changes have been reported in *SLC3A2* transcript in PTPRJ-overexpressing cells compared to controls, thus suggesting that post-translational modifications could account for CD98hc protein downregulation.

To this purpose, we investigated the CD98hc ubiquitylation status following to PTPRJ overexpression. As shown in Figure 4B, PTPRJ induced ubiquitylation compared to control. To determine whether PTPRJ promotes CD98hc degradation, A549 cells infected with Ad *PTPRJ* at MOI50 were treated with MG132, a proteasome inhibitor; cell extracts were purified twenty-four, forty-eight, and seventy-two hours after infection. As expected, Ad *PTPRJ*-transduced A549 cells treated with MG132, did not undergo CD98hc protein levels decrease compared to MG132-untreated cells transduced with Ad *PTPRJ*, where we observed a time-dependent reduction of CD98hc protein levels. These data strongly indicate that PTPRJ induces CD98hc proteasomal-mediated degradation (Figure 4C). In order to validate the PTPRJ-dependent effect on the ubiquitin-mediated CD98hc degradation, we also performed a protein stability assay in the presence of cycloheximide. As expected on the basis of the ubiquitination assay (Figure 4B), the degradation of CD98hc appeared steadily and significantly increased in the presence of virus-dependent *PTPRJ* overexpression, compared to its orthologic control (Figure 4D).

### Simultaneous PTPRJ overexpression and CD98hc knockdown inhibits cell growth and migration of A549 cancer cells and effectively triggers their apoptosis

**Figure 5:**
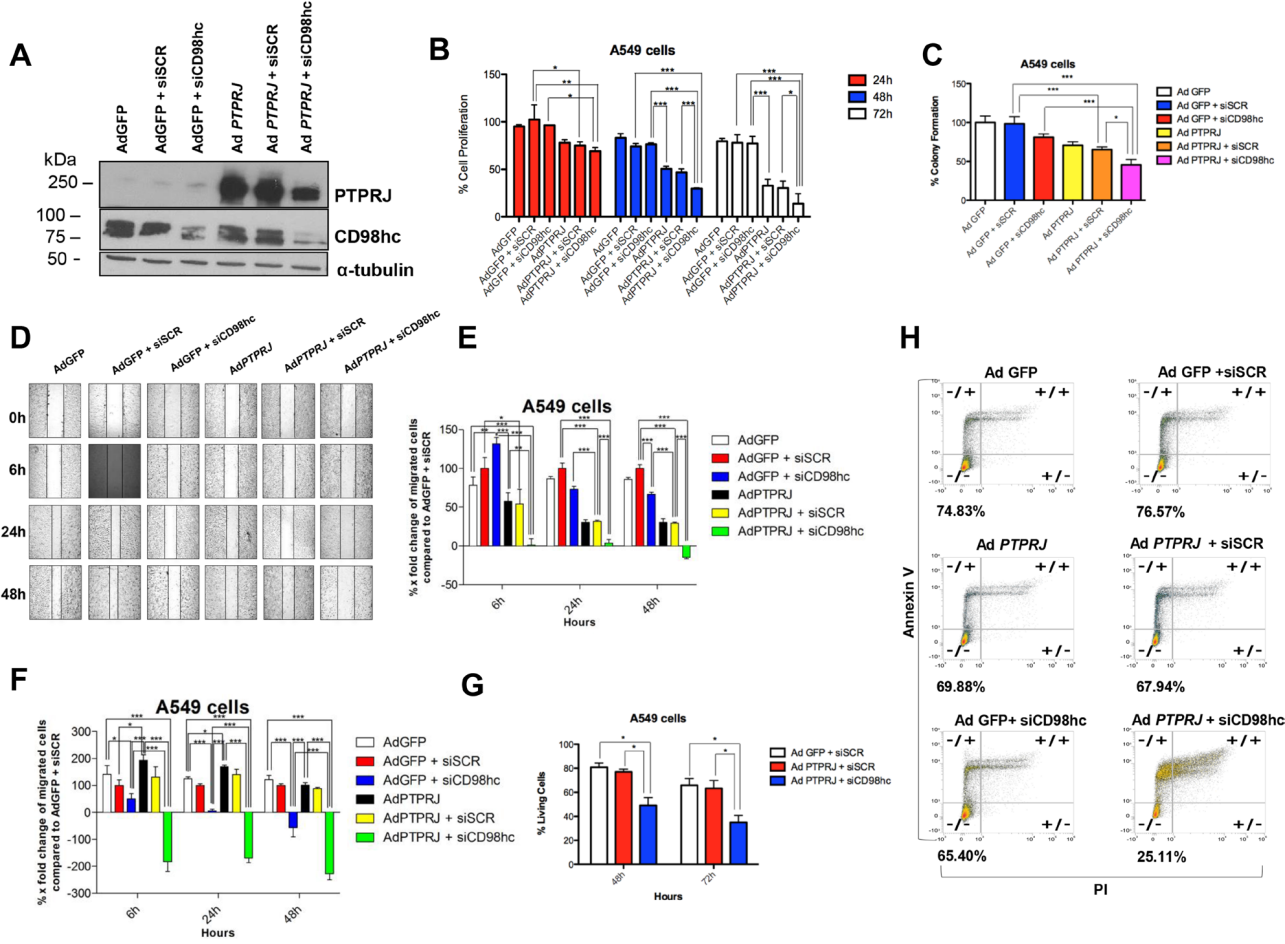
PTPRJ overexpression synergizes with CD98hc silencing in decreasing proliferation and migration of cells A549 and effectively triggers their programmed cell death (**A**) CD98hc silencing was combined to PTPRJ overexpression. A549 cells were transfected with siCD98hc (400 nM) and after 6 h transduced with Ad *PTPRJ* (MOI50). After 72 h cells were lysed and extracted proteins loaded on polyacrylamide gel, a Western blot analysis was performed using cd98hc and PTPRJ antibodies. α-tubulin was used to normalize protein loading. (**B**) Proliferation of A549 cells transfected with siCD98hc (400 nM) and, six hours later, transduced with Ad *PTPRJ* at MOI50. Cell viability was measured twenty-four, forty-eight, and seventy-two hours after infection by CellTiter-Glo^®^ Luminescent assay; values were normalized to Ad GFP. ^*^P < 0.05 value, analyzed by ANOVA, was compared to control. (**C**) PTPRJ overexpression along with CD98hc downregulation significantly decreases A549 cell colony growth. The graph shows the percentage of the number of colonies formed on the culture plate twelve days after treatment. ^*^P < 0.05. (**D, E**) Migration of A549 cells transfected with siCD98hc (400 nM) and, after six hours following transfection, transduced either with Ad *PTPRJ* or Ad GFP at MOI50. Wound-healing assay was performed by measuring the distances between the points of the scratch after six, twenty-four and forty-eight hours following the wound formation. X-fold changes of each sample, compared to its corresponding at *t* = 0 h, were calculated. ^*^P < 0.05 compared with negative control (Ad GFP + siSCR), analysed by ANOVA. (**F**) The same experimental design was also applied to perform a cell migration assay in serum-free conditions. (**G, H**) PTPRJ overexpression with CD98hc silencing increases A549 cell apoptosis. A549 cells were transfected with siCD98hc (400 nM) or siSCR (400 nM) and, after six hours following transfection, transduced with either Ad *PTPRJ* or with Ad GFP at MOI20. Annexin assay was performed forty-eight (panel g) and seventy-two hours (panels g and h) after the treatment; the percent of living cells is reported in both panels. ^*^P < 0.05 compared to negative control (Ad GFP + siSCR), was analysed by ANOVA.

To investigate the effects of PTPRJ and CD98hc interaction on cell proliferation, we either transduced A549 cells with Ad *PTPRJ* or silenced CD98hc through specific siRNA (Figure 5A). While CD98hc knockdown only slightly impaired cell proliferation compared to mock-transfected cells, PTPRJ overexpression significantly reduced A549 cell number; however, the combination of both PTPRJ overexpression and CD98hc knockdown resulted in a further decrease of cell proliferation compared to PTPRJ overexpression alone (Figure 5B). We also examined, by clonogenic assay, the anticancer properties of PTPRJ overexpression in combination with siCD98hc; this treatment significantly decreased A549 ability to form colonies compared to controls (Figure 5C). In addition, by using the same experimental design reported above, we also performed two wound-healing assays by observing either A549 cells cultured under conventional conditions (Figure 5D and 5E) or, on the other hand, in serum-free culture media (Figure 5F). Consistently, PTPRJ overexpression plus CD98hc knockdown resulted in a decrease of cell motility compared to controls in both experiments (Figure 5D, 5E, and 5F).

To assess whether A549 cells undergo programmed cell death after *PTPRJ* adenovirus-mediated overexpression and CD98hc knockdown we performed an annexin/PI assay by flow cytometry. Forty-eight (Figure 5G), and seventy-two hours after treatment (Figure 5G and 5H), we observed that CD98hc siRNA plus PTPRJ overexpression triggered apoptosis in a much more effective way than controls. In fact, while CD98hc silencing and Ad *PTPRJ* overexpression alone showed a low percentage of apoptotic cells (~10% and ~5%, respectively), the combination of both resulted in a dramatic increase in the number of apoptotic cells (more than 50% of dead cells; see Figure 5G and 5H).

### Poor overall survival of non-small cell lung cancer (NSCLC) patients displays low *PTPRJ* and high *SLC3A2* gene expression

**Figure 6:**
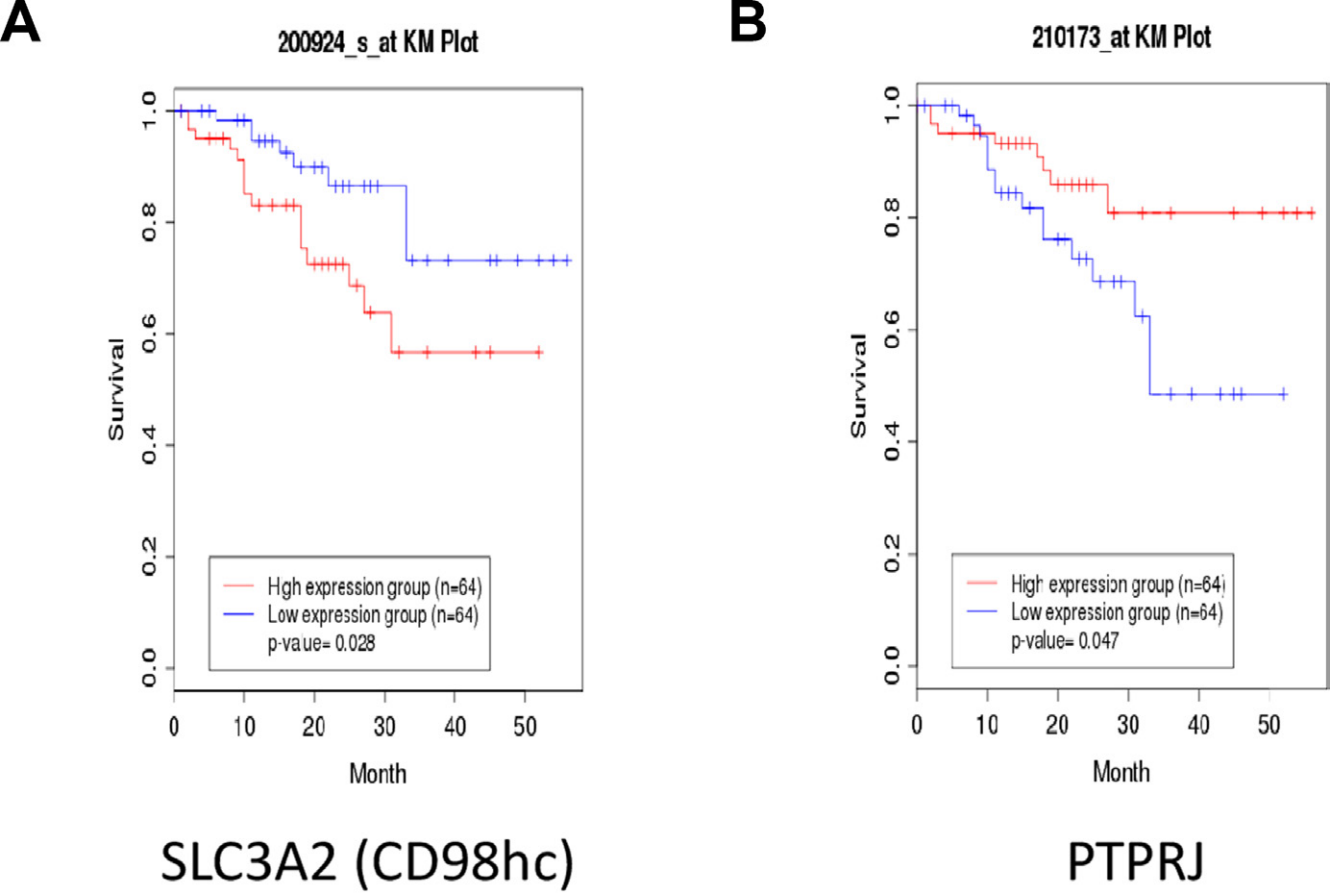
Non-small Cell Lung Cancer (NSCLC) patients with poor outcome show enhanced *SLC3A2* and decreased *PTPRJ* expression levels Public database canEvolve (www.canevolve.org) was interrogated to assess both *SLC3A2* and *PTPRJ* gene expression; *SLC3A2* was found to be increased in NSCLC patients with poorer outcomes (panel **A**) while, in the same patients, *PTPRJ* gene expression decreased (panel **B**).

To understand if our findings might have a clinical significance, we interrogated the clinical public database canEvolve (www.canevolve.org): non-small cell lung cancer patients who experienced the longest survival rate showed lower *SLC3A2* and higher *PTPRJ* gene expression compared to patients with dismal prognosis that, in turn, exhibited the highest and the lowest *SLC3A2* and *PTPRJ* gene expression, respectively (Figure 6A and 6B).

## DISCUSSION

As widely reported, the targeting of disease-associated molecules has become very important for the development of new drugs that can interfere with their aberrant activity; this approach has already provided new therapies for disease treatment [34]. Protein tyrosine phosphatases are very interesting targets for the development of novel drugs, as aberrant tyrosine phosphorylation is involved in human diseases including cancer [35–37]. It was extensively demonstrated that PTPRJ negatively modulates mitogenic signaling by dephosphorylating a number of downstream proteins including receptor tyrosine kinases [18–21]. These findings reinforce the idea of PTPRJ as a target for triggering antiproliferative signals. Indeed, although the use of two biological PTPRJ ligands recently discovered (syndecan-2 and TSP-1) [16, 17] appears of difficult translation into clinics, both CD148 monoclonal antibodies [13] and synthetic PTPRJ agonist peptides seem promising tools for the development of new anticancer drugs [14, 15].

However, as it is apparent from a large body of literature that PTPs are not only involved in cell proliferation [36, 38–40], it is our opinion that we are still very far from understanding all biological processes regulated by PTPRJ. This consideration forced us to seek for novel candidate PTPRJ-interacting proteins. Insights from this approach could expand, not only our knowledge about PTPRJ signaling, but also supply opportunities for the development of novel drugs of easier application compared to CD148 monoclonal antibodies and PTPRJ peptides.

Here, we validated CD98hc as an interesting PTPRJ partner. It is well known that CD98hc has two biochemical functions: a) it binds to cytoplasmatic tails of integrin-β chains mediating adhesive signals that control cell spreading and proliferation and b) it forms a heterodimeric complex with specific light chains of amino acid transporters such as LAT1 and LAT2 with nutrient function [41]. Recent studies have demonstrated LAT1 overexpression in lung cancer, esophageal carcinoma and ovarian tumors [42]; moreover the overexpression of LAT1 is a key factor for the prediction of poor prognosis in non-small cell lung cancer and tends to increase from low-grade to high-grade neuroendocrine tumors of the lung [42, 43]. Since LAT1 light chain requires a covalent association with CD98hc for its functional expression in plasma membrane [44], the heavy chain could play a key role in tumor development and progression. In fact, it is reported that high CD98hc immunoreactivity correlates with high LAT1 expression [45]; moreover, overexpression of LAT1 light chain and CD98hc plays an important role in the progression and metastasis of several human neoplasms since the level of both chains increase markedly in the metastatic sites compared with the primary sites of the tumor [46]. On the contrary, CD98hc is not involved in the intrinsic transport properties of LAT1, as recently demonstrated [47]. In the present study we demonstrate that PTPRJ both dephosphorylates and promotes CD98hc ubiquitylation and proteasome degradation and accordingly, that CD98hc protein downregulation is dependent on PTPRJ overexpression in lung cancer cells. It is well established that initial signals could result in diverging effects; in this specific case, this applies to PTPRJ in terms of protein stabilization or degradation. In fact, while the cell cycle inhibitor p27^kip1^ protein is protected from degradation by PTPRJ-mediated proteasome inhibition, here we demonstrated that the CD98hc oncoprotein is addressed by PTPRJ activity to proteasome-mediated degradation, with a clear-cut effect on its stability. As the discovery of small molecules able to selectively control the degradation of proteins involved in cancer represent an intriguing path for the generation of novel and potentially more effective anticancer drugs [48, 49], these findings strongly and further encourage the development of drugs activating PTPRJ and/or inhibiting CD98hc [50]; this idea is also supported by our results demonstrating that PTPRJ overexpression, combined to CD98hc-silencing, impaired both cell proliferation and migration as well as effectively triggered apoptosis of lung cancer cells to a much higher extent compared to controls. Interestingly, an anticancer therapy targeted to CD98-specific through a human monoclonal antibody, IGN523, elicited a strong antibody-dependent cell-mediated cytotoxicity (ADCC) activity resulting in cell death. Interestingly, IGN523 is currently being evaluated in a Phase I clinical trial for acute myeloid leukemia [51].

The relationship between PTPRJ and CD98hc is interesting also from a clinical point of view since non-small cell lung cancer (NSCLC) patients who experienced the longest survival presented lower expression of CD98hc and higher expression of PTPRJ genes compared to patients with poor outcome (www.canevolve.org). These data suggest that both *PTPRJ* and *SLC3A2* transcripts could potentially be considered as interesting biomarkers of lung cancer progression.

However, we consider the PTPRJ-CD98hc interaction as the very beginning of a number of mechanistic studies based on the protein network involving PTPRJ itself. As an example, it has been reported that CD98hc interacts with CD147, a member of the superfamily of immunoglobulin, that coordinates the transport of both amino acids and lactate in cancer cells [52, 53]. Interestingly, in our raw list of proteins potentially interacting with PTPRJ we also found CD147, even though the enrichment in PTPRJ-His6 observed by mass spectrometry was below the significance threshold (Supplementary Table 1); this information, validated by preliminary experiments of co-immunoprecipitation indicating that PTPRJ interacts with CD147, stimulates further investigations in order to shed lights about the role of the PTPRJ/CD98hc/CD147 complex also in the energetic metabolism of cancer cells.

In conclusion, the development of drugs aimed to both trigger PTPRJ activity and inhibit CD98hc signaling in combination, could represent an intriguing opportunity for the treatment of cancer patients with poor prognosis, based on the simultaneous targeting of several hallmarks important in the biology of a cancer cell [54], including proliferation, apoptosis, angiogenesis, and energetic metabolism.

## MATERIALS AND METHODS

### Cell culture, transfection and infection experiments

A549 lung cancer cells were cultured in RPMI medium 1640 supplemented with 10% FBS (Sigma-Aldrich) and were maintained at 37° C in a humidified atmosphere of 5% CO_2_. Transfections were made with Lipofectamine 2000 (Invitrogen) by following the manufacturer's instructions; 3.5 × 10^5^ A549 cells were seeded in 6-well plates and transfected with 100 pmol of either CD98hc-specific or scrambled siRNAs (Life Technologies). Infections were carried out by infecting 3.5 × 10^5^ A549 cells with Ad *PTPRJ* at multiplicity of infection (MOI) 50. The generation of recombinant Ad *PTPRJ* and Ad *PTPRJ*-His6 was performed as previously described [14].

### Identification of PTPRJ-binding proteins by mass spectrometric analysis

PTPRJ-His6 recombinant protein was overexpressed in A549 by infecting 5 × 10^7^ cells with Ad *PTPRJ*-His6 at MOI30. Three days after infection, cell lysis was performed with Mem-PER Eukaryotic Membrane Protein Extraction Kit (Pierce) to enrich mature transmembrane proteins. Mature recombinant PTPRJ-His6 protein and its candidate interactors were purified through MagneHis Protein Purification System (Promega) following the manufacturer's protocol. Beads were extensively washed with HEPES 100 mM with added NaCl 500 mM (pH 7.5) to remove unbound proteins. Proteins were finally eluted from the beads by using 100 mM HEPES and 500 mM imidazole elution buffer (pH 7.5). The eluates (50 μL) from PTPRJ-His6 and control (wild-type PTPRJ) were mixed with 100 μL of Tris buffer (150 mM, pH 8); proteins were reduced by DTT (100 mM, 15 μL) and alkylated by iodoacetamide (200 mM, 18 μL). After quenching excess iodoacetamide by the addition of 3 μL of DTT solution, 400 ng of Proteomics grade trypsin (Sigma) were added, and the enzymatic reaction was allowed to proceed overnight at 37° C with shaking.

The peptide mixtures were acidified with trifluoroacetic acid (TFA, final concentration 0.5%) and purified by solid phase extraction (SPE cartridges HLB, 1 cc, Waters). Concentrated eluates were labeled by dimethyl labeling, mixed, purified and fractionated by strong cation exchange (SCX) StageTips as described in Varano *et al*. [55] PTPRJ-His6 sample was labeled “heavy”, whereas the control was labeled “light”. The SCX fractions (*n* = 6) were evaporated to dryness and reconstituted in 8 μL of 2% acetonitrile/0.1% formic acid. Mass spectrometry analysis was performed on a Q-Exactive Hybrid Quadrupole-Orbitrap Mass Spectrometer coupled online to an Easy nano-LC1000 system (Thermo Fisher Scientific, Germany) as described [55] using a TOP-12 data-dependent method. Raw data were processed by Proteome Discoverer 1.4 (Thermo Fisher Scientific, Germany) using the Sequest algorithm and searched against the Uniprot Human reference proteome database (March 2015, 67948 sequences). The search criteria were set as follows: enzyme trypsin, maximum two missed cleavages, Dimethyl (Any N-Terminus), Dimethyl (K), Carbamidomethyl (C) as static modifications (dimethyl modifications were set to either “light” (L) or “medium” (M) in two parallel searches), Oxidation (Met) as dynamic modification, MS tolerance 15 ppm, MS/MS tolerance 0.02 Da. A FDR of 1% at the peptide level was estimated using the Target Decoy PSM Validator node available in Proteome Discoverer. A minimum of two peptide identifications per protein was required.

Protein quantification was performed in Proteome Discoverer 1.4 using the following parameters: minimum quantification value threshold = 0, missing quantification values replaced with minimum intensity, maximum allowed fold change = 100, ratios above maximum allowed fold change for quantification: not used, % co-isolation excluding peptides from quantification = 100, quantification on all peptides. Normalization of M:L was performed on PD, using the protein median M:L value.

Permutation statistical analysis of the peptide ratios was performed using Quantitative Proteomics *p*-value Calculator (http://qppc.di.uq.edu.au/) [56]. Number of permutations was set to 10,000. Proteins increased in the PTPRJ-His6 pull-down with *p*-values < 0.01 were considered significantly enriched. Gene ontology (GO) and protein network analysis of the significantly altered proteins was performed using STRING v10.5 (https://string-db.org/) [57]. The interaction score was set to 0.4; protein interactors were query proteins only.

### Co-immunoprecipitation and Western blot analysis

Total proteins were extracted with Igepal CA-630 lysis buffer containing 50 mMTris-HCl pH 7.5, 150 mMNaCl, 0.5% Igepal CA-630, 1 mM Na_3_VO_4_, 25 mMNaF, 2 mM PMSF, 8 nMAprotinin; enriched plasma membrane proteins, used for both mass spectrometry and immunoprecipitations analyses, were obtained using Mem-PER Eukaryotic Membrane Protein Extraction Kit (Pierce). For CD98hc immunoprecipitation, cell extracts were incubated overnight at 4° C with CD98hc antibody and then overnight with 20 μl of protein A/G Plus-Agarose (Santa Cruz Biotechnology). Pellets were washed five times with 1 ml of lysis buffer, resuspended in Laemmli sample buffer 2× and subjected to SDS-poly-acrylamide electrophoresis (SDS-PAGE) [58]. For PTPRJ immunoprecipitation, MagneHIS Protein Purification System (Promega) was used as described above. For Western blot analysis, proteins (50 μg) were loaded and separated on polyacrylamide gels and transferred to nitrocellulose filter membranes. Membranes were blocked in 5% non-fat dry milk, incubated with primary PTPRJ (R&D Systems), CD98hc (Cell Signaling Technology), p-Tyr, Ubiquitin, γ-tubulin, α-tubulin and GAPDH antibodies (Santa Cruz Biotechnology), detected by the appropriate secondary antibodies (Santa Cruz Biotechnology) and revealed by enhanced chemiluminescence (ECL; Amersham Inc.).

### Immunofluorescence

A549 cells were fixed with 4% paraformaldehyde at 4° C for 30 min, rinsed with distilled H_2_O and then blocking in 1% BSA was performed for 1 h at room temperature. Cells were subsequently incubated for 2 h at room temperature with PTPRJ antibody (1:40), rinsed with distilled H_2_O and permeabilized with ice-cold 100% MeOH for 15 min at –20° C. Afterwards, cells were washed and blocking was performed again. Cells were, then, incubated overnight at 4° C with CD98hc antibody (1:800). After being washed with PBS two times, PE-labeled anti-mouse IgG (1:25) and Alexa Fluor 633-labeled anti-rabbit (1:500) (Thermo-Fisher Scientific) were added as secondary antibodies and DAPI (1:1000) for nuclei staining. Immunofluorescence was visualized with a laser-scanning confocal imaging system.

### Cycloeximide assay: protein stability

Treatment with cycloeximide (CHX) 100 μg/mL was performed on 5 × 10^5^ A549 cells, 48 hrs after infection with either Ad *PTPRJ* (MOI50) or Ad GFP, as a control. The treatment was added at different time point (0, 30 minutes, 1 hour, 2 hours). Western blotting analysis of total proteins (60 μg) was carried out by PTPRJ (R&D System) and CD98hc (Cell Signaling Technology) antibodies. *Bona fide* protein degradation was assayed by means of Pdcd4 (Santa Cruz) protein stability, as positive control. Equal loading was verified by tubulin (Sigma) and HDAC (Sigma).

### Quantitative real time PCR

A549 cells were seeded in 100 mm culture dishes and, after 24 hours, transduced by a recombinant Adenovirus carrying a *PTPRJ* cDNA at a MOI50. RNA extraction was carried out with miRNeasy Mini Kit™ (Qiagen, Valencia, CA, USA), following the manufacturer's instructions, and total RNA was quantified with a spectrophotometer. Total RNA samples (250 ng) were retro-transcripted using the High Capacity RNA-to-cDNA Kit (Applied Biosystems, Foster City, CA, USA), following the manufacturer's instructions. Five hundred nanolitres of cDNAs were amplified by real-time PCR with Promega SYBR green kit and 5 pmol of primers in a total volume of 25 μL. The primers used were: 5′-TCCCAGAATGCCGAGATGAT-3′ (forward to *SLC3A2*), 5′-GCTCCACCTCCTTCATATCC-3′ (reverse to *SLC3A2*), 5′-GTATTATCATTGGTGGCTTGTTC-3′ (forward to *PTPRJ*), 5′-CATCTCCGTGGTGGTGAC-3′ (reverse to *PTPRJ*) and 5′-AGGCAGGTGTTCAAATCAT CC-3′ (reverse for short form of *PTPRJ*). Real-time PCRs were performed in a BioRad iQ™ 5 tool in the following conditions: initial denaturation step at 95° C for 3 min, followed by 40 cycles of 10 s at 95° C and 1 min at 57° C. Specificity of PCR products was checked by melting curve analysis and gel electrophoresis. Values were normalized to HPRT RNA levels, ^*^P < 0.05.

### Cell survival assay

1 × 10^4^ A549 cells were seeded in 96-well plates; cells were first transfected with 5 pmol of siCD98hc and six hours later transduced with Ad *PTPRJ* at MOI50. Cell proliferation was measured out twenty-four, forty-eight, and seventy-two hours after treatment through CellTiter-Glo Luminescent Cell Viability assay (Promega) following the manufacture's protocols. The results were expressed as percent variation in the number of viable cells treated with CD98hc siRNA plus Ad *PTPRJ* compared with control Ad GFP plus scrambled siRNA treated cells.

### Clonogenic assay

Briefly, 5 × 10^5^ A549 cells were seeded per well in a 6-well plate. The next day cells were transfected with CD98hc siRNA (100 pmol) and six hours later transduced with Ad *PTPRJ* at MOI50, and incubated overnight. Following incubation, cells were harvested by trypsinization and counted; 300 cells then were seeded in 6-well plate and were incubated at 37° C. Twelve days later, the medium was removed and colonies were fixed and stained with cristal violet. Colony counting was performed by three different investigators.

### Wound-healing assay

To examine cell migration, 3.5 × 10^5^ A549 cells were seeded in a 6-well plate; the following day, cells were transfected with 100 pmol of CD98hc siRNA and infected with Ad *PTPRJ* at MOI50; twenty-four hours later, cell layer in the dishes was scratched using 200 μl plastic pipette tips as previously described [59]. The closure of wounded area was measured six, twenty-four and forty-eight hours later by using a motorized inverted fluorescent microscope coupled with an high-sensitivity EM-CCD camera (Leica). The same experiment was performed in serum free conditions.

### Apoptosis assay

A549 cells were cultured in 6-well plates; the day after, cells were transfected with 100 pmol of CD98hc siRNA and six hours later transduced with Ad *PTPRJ* at MOI20; twenty-four, forty-eight, and seventy-two hours later cells were analyzed by flow cytometry. Apoptosis was evaluated by FACS analysis following Annexin/V-7AAD staining (BD Pharmigen). Annexin V^+^/PI^-^ cells meant early apoptotic cells while Annexin V^+^/PI^+^ cells meant late apoptotic cells.

### Statistical analysis

Graphical analysis was performed by using GraphPad Software Prism 5. One-way analysis of variance (ANOVA) test, followed by Bonferroni post-test (for multiple comparisons), was used to detect the statistical significance. Differences in the results were considered significant with *p*-values < 0.05. Statistical analysis for proteomic data was previously described in the section “Identification of PTPRJ-binding proteins by mass spectrometric analysis”.

## SUPPLEMENTARY MATERIALS AND TABLES






