# Supplementary material for: The receptor protein tyrosine phosphatase PTPRJ negatively modulates the CD98hc oncoprotein in lung cancer cells
Source: Oncotarget. 2018 May 4;9(34):23334–48. doi: 10.18632/oncotarget.25101 (PMC5955124; doi:10.18632/oncotarget.25101)
Supplement: Supplementary file 1 [file oncotarget-09-23334-s001.pdf]

# The receptor protein tyrosine phosphatase PTPRJ negatively modulates the CD98hc oncoprotein in lung cancer cells

## SUPPLEMENTARY MATERIALS

**Supplementary Table 1: List of proteins identified by a minimum of 2 high confidence peptides.** See Supplementary_Table_1.

**Supplementary Table 2: Bioinformatic analysis of the proteins enriched in PTPRJ-His6 pull-down, performed using STRING.** See Supplementary_Table_2.
